# Real World Evidence in Medical Cannabis Research

**DOI:** 10.1007/s43441-021-00346-0

**Published:** 2021-11-08

**Authors:** Rishi Banerjee, Simon Erridge, Oliver Salazar, Nagina Mangal, Daniel Couch, Barbara Pacchetti, Mikael Hans Sodergren

**Affiliations:** 1 Department of Surgery and Cancer, Imperial College London, London, UK; 2 Sapphire Medical Clinics, UK Medical Cannabis Registry, London, UK; 3 The Centre for Medicinal Cannabis, 18 Hanway Street, London, W1T 1UF UK; 4 Curaleaf International, London, UK; 5 Division of Surgery, Department of Surgery & Cancer, Imperial College London, St Mary’s Hospital, Academic Surgical Unit, 10th Floor QEQM, South Wharf Road, London, W2 1NY UK

**Keywords:** Cannabis, Cannabidiol, Delta(9)-tetrahydrocannabinol, Medicinal Cannabis, Cannabis-based medicinal products, Real world evidence

## Abstract

**Background:**

Whilst access to cannabis-based medicinal products (CBMPs) has increased globally subject to relaxation of scheduling laws globally, one of the main barriers to appropriate patient access remains a paucity of high-quality evidence surrounding their clinical effects.

**Discussion:**

Whilst randomised controlled trials (RCTs) remain the gold-standard for clinical evaluation, there are notable barriers to their implementation. Development of CBMPs requires novel approaches of evidence collection to address these challenges. Real world evidence (RWE) presents a solution to not only both provide immediate impact on clinical care, but also inform well-conducted RCTs. RWE is defined as evidence derived from health data sourced from non-interventional studies, registries, electronic health records and insurance data. Currently it is used mostly to monitor post-approval safety requirements allowing for long-term pharmacovigilance. However, RWE has the potential to be used in conjunction or as an extension to RCTs to both broaden and streamline the process of evidence generation.

**Conclusion:**

Novel approaches of data collection and analysis will be integral to improving clinical evidence on CBMPs. RWE can be used in conjunction or as an extension to RCTs to increase the speed of evidence generation, as well as reduce costs. Currently, there is an abundance of potential data however, whilst a number of platforms now exist to capture real world data it is important the right tools and analysis are utilised to unlock potential insights from these.

## Background

Cannabis-based medicinal products (CBMPs) are a collective term to describe *a preparation or other product that contains cannabis or its derivatives for medicinal use in humans* [1]. There are significant barriers to the integration of CBMPs within treatment pathways including ongoing stigma, cost, education, complex pharmacology and a paucity of evidence to inform international and national guidelines [2, 3]. Limited evidence, does, however, support the role of CBMPs in conditions such as chronic pain, neurological disorders, and psychiatric disease [4]. There is also growing evidence of side effects and how the severity and incidence of side effects may differ between patients [4]. The quality of evidence, however, is often insufficient in the opinion of insurers, regulators, and guideline bodies [5].

The National Institute for Health and Care Excellence in the UK has only recommended licensed CBMPs for a limited range of indications [6]. Changes to scheduling as recommended by the World Health Organisation, and within individual countries, recognises the potential medicinal value of cannabis and removes barriers for clinical and research use [1, 7]. However, widespread stigma, complex pharmacology, funding, and challenges in sustaining adequate supply of consistent products continue to act as barriers for clinical research.

Randomised controlled trials (RCTs) are necessary and should continue to be the standard against which medical evidence is upheld. However, they are expensive, time consuming and subject to their own limitations [8]. Whilst these are awaited, there is a requirement to generate evidence of potential benefits and harms to inform policy and clinical practice.

## Barriers to Controlled Clinical Trials for Medical Cannabis

RCTs are not infallible—they are expensive and time consuming. Globally $100 billion USD is spent on biomedical research [9]. In the UK, the National Institute for Health Research (NIHR) provides £80 million GBP in funding for clinical trials [10]. Yet, their narrow scope can lack ecological validity to real-world circumstances and therefore lack generalisability in more diverse populations. There are also specific barriers to conducting RCTs using CBMPs.

### Complex Pharmacology

In addition to cannabidiol (CBD) and (−)-trans-Δ^9^-tetrahydrocannabinol (THC) there are over 140 cannabinoids, as well as flavonoids, terpenes, and other compounds within the flower of different cannabis plants [8]. These can each potentially affect the clinical outcomes observed between CBMPs due to their individual and collective effects [11]. The concentrations of each compound are influenced by the genetics and environment each plant is grown in producing a distinct chemical profile. The result of a clinical trial for one finished pharmaceutical product, therefore, cannot be extrapolated to all CBMPs, due to their heterogeneity. However, current evidence reviews often fail to account for this [12, 13].

The route of administration further affects the pharmacokinetics of CBMPs and the associated outcome of any trial. CBMPs can be administered sublingually, trans-dermally, via inhalation, or orally [14]. This subsequently affects the distribution, biotransformation and elimination of active compounds. Heat exposure and vaporisation of dried flower or extracted oils changes the underlying phytocannabinoid composition compared to the original unprocessed dry flowers, increasing the proportion of decarboxylated cannabinoids [15, 16]. Assessment of efficacy using RCTs in isolation will therefore ultimately fail to identify the most appropriate CBMP for each clinical scenario [17].

### Placebo-control

An appropriately blinded assessment against placebo or active therapy is the optimal design for RCTs. It has been difficult to identify a placebo that cannot be distinguished against an active CBMP according to absence of both vasoactive and psychoactive effects, as well as the typical aroma associated with cannabis [15]. This presents a challenge to adequate blinding.

### Cost

Production methods and import costs mean that CBMPs are typically expensive, adding further to high research costs [18]. Research has therefore focused on compounds under patent as opposed to generic CBMPs where research outcomes fail to provide a similar return on investment for licensed producers and pharmaceutical companies. Historically, clinical trials on CBMPs were funded privately, which may be associated with potential reporting biases [19].

RCTs are possible with CBMPs; however, the above issues present legitimate challenges. In many chronic diseases there is a need for novel therapeutics and CBMPs are therefore being utilised based on best available evidence. Due to the challenges in developing CBMPs through a traditional drug development pipeline, the exploration of its utility should not be limited to traditional methods. It is important that we capture a suite of real-world evidence (RWE) to inform prescribing guidelines, regulations, and clinical trials. By leaning on RWE there is an opportunity to improve the quality and design of RCTs and clinical evidence in general, via a top-down approach [20].

## Real World Evidence

RWE is defined as *evidence derived from health data sourced from non-interventional studies, registries, electronic health records and insurance data* as opposed to the highly controlled setting of RCTs [21]. There is an abundance of this unstructured data, however, the necessary frameworks and governance are needed for the application of this data [22]. It is currently used extensively to monitor post-approval pharmacovigilence [23]. There is clear evidence of benefit in using population-based data to detect safety events associated with specific medications to implement restrictions to reduce harm [21].

Consistent use of RWE to aid regulatory decision making is yet to be normalised, but the promise is apparent [21]. Recently, regulator-supported initiatives have highlighted the desire to incorporate RWE into licensing and guidelines, developing a framework which can incorporate its insights into decisions regarding safety and effectiveness [21, 22]. It is important that studies standardise their methodology according to those set out by regulatory authorities to ensure research has the greatest impact [21, 22]. Moreover, they should seek to directly address questions set out by governing bodies as areas where there is insufficient research [24].

## Types of Real-World Evidence for Medical Cannabis

NHS England and NHS improvement published a review on the barriers to accessing CBMPs in the UK [3]. Their recommendations included the need for the collection of structured data, and the development of methods to further support the generation of new evidence, for patients who cannot enrol onto relevant RCTs.

RWE is already being incorporated into the scientific literature on cannabis (Table 1). Early examples utilised state-level records to examine the effects of cannabis laws on opioid misuse. Subsequently there have been examples of online and self-administered survey tools analysing national outcomes. More recently there has been a focus on collecting evidence from clinical registries and databases with evidence generated from patient-reported outcome measures and long-term pharmacovigilance.

**Table 1:** Examples of Real World Evidence Generation for Cannabis-Based Medicinal Products

Regional/National Survey Studies	
Ware et al. [31]	Analysis of self-administered questionnaire study conducted in the UK between 1998 and 2002
Sexton et al. [32]	Anonymous online survey analysing medical cannabis users and which conditions they use it to treat focusing on patient perception of efficacy, and physical and mental health
Lucas et al. [33]	Patients who were registered to a federally authorized licenced producer in Canada were requested to fill out a self-administered survey
Lintzeris et al. [34]	Cannabis As Medicine Survey (CAMS-18) was an online, anonymous survey given to patients recruited mainly via social media in Australia. The consequent analysis looked at patterns of use and perspectives on medical cannabis
Government records analysis	
Bachhuber et al. [35]	Time series analysis of medical cannabis laws and state-level death certificate data in the United States from 1999 to 2010
Piper et al. [36]	Analysed data from drug arrest data from the Maine Diversion Alert Program regarding diversion of prescription medications subject to scheduling, such as cannabis
Vigil et al. [37]	Retrospective cohort study looking at the association between enrolment in the New Mexico Medical Cannabis Program and opioid prescription use
Analysis of clinic/dispensary data	
Bonn Miller et al. [38]	Cross sectional study of medical cannabis use from a cannabis dispensary in California. The study highlighted perceived benefits in conditions where medical cannabis had not yet been licensed
Gulbransen et al. [39]	An audit of all patients who presented to a Cannabis Care clinic in New Zealand. This study highlighted the anxiolytic effects of cannabidiol
Rapin et al. [40]	A retrospective observational study looking at patients who had been prescribed CBD treatment from data collated from a network of clinics
Analysis of registries	
Habib et al. [27]	Looked at the efficacy of medical cannabis for treatment of fibromyalgia. It used data from two hospital registries. The relevant patients also filled out a questionnaire
Ueberall et al. [41]	Evaluated RWD collected from the German Pain e-Registry. It looked at the efficacy and tolerability of an oromucosal spray composed of THC: CBD. The registry was developed by the Institute of Neurological Sciences on behalf of the German Pain Association
Mahabir et al. [42]	Analysed data from a registry which contained data on some medical cannabis evaluation clinics in the United States. Database owned by CB2 Insights
Databases	
Quebec Cannabis Registry (Canada) [43]	Collated clinical data collected from users of dried medical cannabis with 6000 participants in Quebec
Medical Cannabis Real World Evidence [44–46]	A Canadian, prospective, non-interventional, observational study led by the University Health Network in Toronto. It aims to explore the benefits of medical cannabis in an observational setting for adults with conditions such as chronic pain, anxiety or depression. The study will evaluate the efficacy and safety of a range of CBMPs selected by the patients themselves after assessment by a clinician. The study currently aims to recruit 2000 participants, with completion due in 2022
UK Medical Cannabis Registry (UK) [47, 48]	Captures clinical data on prescribed CBMP formulations, adverse events and patient-reported outcome measures across a myriad of conditions at defined timepoints. The principal aim is to generate evidence demonstrating the effect of CBMPs on health status or health-related quality of life and generate more accurate calculations of adverse effects. Published early experience of improvement in health-related quality of life, sleep and anxiety outcomes across a range of conditions
Project Twenty21 (UK) [49, 50]	Registry platform for data collection from patients provided prescriptions by licensed producers at subsidised cost for enrolment. Limited to six specific conditions, openly aiming to change healthcare funding decisions. Aims to recruit 20,000 patients with initial data analysis due in 2022
Emerald Clinics (Australia) [51–53]	Real world evidence platform utilising clinical registries and patient generated health data to inform clinical care and aid bespoke drug development. They have utilised this as part of a drug development platform for specific CBMP preparations in psychological disease (EMD-003) and irritable bowel syndrome (EMD-004) in top-down driven drug development
Minnesota Medical Cannabis Programme (US) [54]	This registry collects data on the benefits, risks and outcomes regarding the therapeutic use of medical cannabis. It is run by the Minnesota Department for Health
BfArM (Germany) [55]	A non-interventional survey on the use of these cannabis medicines, which must be completed by any German physician prescribing medical cannabis
CB2 Insights (US) [56]	CB2 Insights is a healthcare technology company that uses real world data to monitor, assess, and generate insights to help improve patient outcomes
Medical Cannabis Pilot Programme (Denmark) [57]	A four-year pilot programme which aimed study patients’ use of medicinal cannabis. The success of the programme has recently led to an extension of the trial period
Italian Medicines Agency (Italy) [58]	In Italy, prescriptions and patient data are registered with the Italian Medicines Agency. In 2019 26,042 medical cannabis prescriptions, attributed to 12,998 patients had been registered
Cannabis a` usage medical programme (France) [59]	Initiated by the French National Agency for Medicines and Health Products Safety, the registry will collate information on efficacy and safety data on medical cannabis. It will supply medical cannabis to 3000 patients over 2 years and aims to examine real life use of therapeutic cannabis
Medical Cannabis Access Programme (Ireland) [60]	A 5-year pilot programme in which certain CBMPs can be prescribed with information on prescribing collected by Ireland’s Health Service Executive

## Comparison of Real-World Evidence and Controlled Clinical Trials

Between these study designs it is important to be aware of potential divergence in reported outcomes. RWE has broader inclusion criteria, accounting for factors like non-standard dosing, and is not limited by scope of disease, thereby improving ecological validity [25]. However, some studies have concluded there is little difference between results obtained via RCTs and observational studies [26]. RWE typically has longer patient follow-up and may consequently capture rare but important adverse effects that are not detected within RCTs. Pharmacovigilance is therefore widely accepted as one of the most important roles of RWE.

RWE can bring further clarity on questions that remain unanswered in RCTs. A recent study utilised anonymised surveys of patients with fibromyalgia who consumed cannabis flower [27]. In addition to reporting positive outcomes on depression and pain the study also reported negative aspects of cannabis consumption, for example driving under the influence (72% of patients) [27]. These are findings which are unlikely to be reported by patients in controlled clinical trials for fear of repercussions, or strict inclusion criteria. It can also be useful in collecting data in rare conditions whereby recruitment to RCTs can be limited by the need for defined trial sites.

RWE can improve the efficiency of clinical trials by generating hypotheses, refining eligibility criteria, and exploring drug development tools. Registries can be used to form an infrastructure to conduct a clinical trial, lowering costs whilst maintaining high evidence quality [28]. In supplemented single arm trials the controls are derived from RWE-data sets, providing the opportunity for patient centric study designs. RCTs can also be augmented with real-world data to increase the size of the control group to increase the power of the study. These study designs are particularly useful for rare diseases where participant recruitment is challenging [29].

## Limitations of Real-World Evidence

RWE, however, does have limits to its utility. There is variation in the quality and provenance of the data stored in electronic medical records [5]. Furthermore, insurance records typically use coding specific for reimbursement purposes and may not provide all clinically relevant information. RWE can require complex statistical expertise to deduce valid conclusions.

Another limitation is the lack of randomisation, controlled variables and internal validity. This can make it more difficult to derive causative mechanisms behind clinical outcomes. However, this is also one of the strengths of these studies, allowing for generalisability to true clinical practice [22]. Treatment assignment based on the physician as opposed to randomisation, creates selection bias and more specifically stigma biases. RCTs, therefore, are still necessary to establish a strong causal relationship between medication and outcomes [30].

## Conclusion

CBMPs are a complex range of pharmaceuticals which pose challenges to traditional pathways of drug development and translation. Development of CBMPs requires novel approaches of evidence collection to address these challenges. RWE can be used in conjunction or as an extension to RCTs to both broaden and streamline the process of evidence generation. Currently, there is an abundance of potential data, however, it is important the right tools and analysis are utilised to unlock potential insights from these.

## Data Availability

Not Applicable.

## Abbreviations

CBD: Cannabidiol
CBMPS: Cannabis-based medicinal products
FDA: Food and Drug Administration
NHS: National Health Service
NIHR: National Institute for Health Research
RCTs: Randomised Controlled Trials
RWE: Real world evidence
SATs: Supplemented single arm trials
***THC*** (−): Trans-Δ^9^-tetrahydrocannabinol
